# The Impact of Thermal Radiation on Maxwell Hybrid Nanofluids in the Stagnation Region

**DOI:** 10.3390/nano12071109

**Published:** 2022-03-28

**Authors:** Nurul Amira Zainal, Roslinda Nazar, Kohilavani Naganthran, Ioan Pop

**Affiliations:** 1 Department of Mathematical Sciences, Faculty of Science and Technology, Universiti Kebangsaan Malaysia, Bangi 43600, Malaysia; nurulamira@utem.edu.my; 2 Fakulti Teknologi Kejuruteraan Mekanikal dan Pembuatan, Universiti Teknikal Malaysia Melaka, Hang Tuah Jaya, Durian Tunggal 76100, Malaysia; 3 Institute of Mathematical Sciences, Faculty of Science, Universiti Malaya, Kuala Lumpur 50603, Malaysia; kohi@um.edu.my; 4 Center for Data Analytics, Consultancy and Services, Faculty of Science, Universiti Malaya, Kuala Lumpur 50603, Malaysia; 5 Department of Mathematics, Babeş-Bolyai University, R-400084 Cluj-Napoca, Romania; popm.ioan@yahoo.co.uk

**Keywords:** stagnation point, Maxwell fluids, hybrid nanofluids, thermal radiation, stability analysis

## Abstract

Previous research has recognised the study of stagnation point flow by focusing Maxwell nanofluid on a stretching sheet surface. Motivated by this research idea, our main objective is to formulate and analyse a new mathematical model of stagnation point flow in Maxwell fluid that highlights the dual types of fluid known as hybrid nanofluids. The effects of thermal radiation and heat transfer are also considered. The partial differential equations (PDEs) are converted into ordinary differential equations (ODEs) via similarity variables that generate similarity solutions. Following that, the bvp4c approach is employed to discover the approximate solutions of the reduced ODEs. The significance of various parameters is graphically presented and considers the physical quantities of interest. A remarkable observation found in this study is the enhancement of the heat transfer rate in Maxwell hybrid nanofluids, which is steadily amplified in contrast to traditional fluids. Indeed, the Maxwell parameter in hybrid nanofluids embarks on a substantial increment of the heat transfer rate. The current study succeeds in establishing more than one solution along the stretching/shrinking sheet. Thus, the stability analysis is conducted to confirm the sustainability of the solutions.

## 1. Introduction

Fluids such as shampoos, ketchup, sugar solution, tomato paste, and soups are classified as non-Newtonian fluids, which cannot be characterised by Newton’s viscosity law. Due to their various rheological properties, these types of fluids are impossible to analyse using a single constitutive relationship. There are numerous non-Newtonian fluid models available, and one of the subgroups is known as the Maxwell model and is considered as rate type fluid. This fluid model is very beneficial for low-molecular-weight polymers. Thus, it has gained a particular reputation among scientists and researchers due to its simplicity, hence permitting investigators to concentrate exclusively on the fluid elasticity impact [1].

Sadeghy et al. [2] performed an investigation of boundary layer flow in two-dimensional stagnation point flow employing the Maxwell fluids. Hayat et al. [3] extended the Maxwell fluids investigation to the stretching surface of stagnation point flow by incorporating magnetic impact using an analytical solution. Abbas et al. [4] added the convection parameter over a vertical stretching surface of stagnation point flow in Maxwell fluids. The study reported that increasing the mixed convection parameter causes the velocity deviation and boundary layer thickness to amplify. Ramesh et al. [5] incorporated the presence of nanoparticles and the Maxwell parameter in stagnation point flow past a permeable surface. Following that, many researchers have become interested in the research of stagnation point flow toward a shrinking sheet in Maxwell fluids. For example, Lok et al. [6] considered the suction parameter in the Maxwell fluid flow and heat transfer model past a shrinking sheet near the stagnation region. They found that dual solutions exist for some values of shrinking and suction parameters for a fixed value of the Maxwell parameter. Jusoh et al. [7] considered three-dimensional Maxwell nanofluids deliberating the convective boundary condition towards a stretching/shrinking sheet. Afterward, Ahmed et al. [8] examined the stagnation point flow of Maxwell nanofluids with heat source/sink past a radially stretching/shrinking disk. Recently, Aziz et al. [9] and Ahmad et al. [10], for instance, examined the study of heat transfer in boundary layer flow of Maxwell hybrid nanofluids due to its capacity to improve the thermal conductivity. 

Radiation properties are one of the most important process parameters in heat and fluid movement throughout a high-temperature thermal system. Thermal radiation is a technique for controlling excess heat emission that has a broad range of uses in the industry. According to Nayak et al. [11], Zainal et al. [12], and Jamaluddin et al. [13], thermal radiation’s impacts on the construction of reliable equipment, nuclear power plants, missiles, satellites, gas turbines, and a variety of complicated conversion systems are indeed crucial. Aliakbar et al. [14] examined thermal radiation with magnetic effects of Maxwell fluid over an accelerating surface. Hayat and Qasim [15] tested the presence of thermophoresis and thermal radiation, employing heat and mass transfer towards a stretching sheet, while Madhu et al. [16] considered the unsteady parameter in Maxwell nanofluids by including the magnetic and radiation effects. Later, Jamshed [17] performed a numerical analysis past an infinite horizontal surface with thermal radiation and demonstrated that the thermal conductivity steadily increases compared to the traditional fluid in Maxwell parameter occurrences.

In the manufacturing industry, transport phenomena within the stagnation region, such as the extrusion process and polymer productivity, are prominent and require ongoing improvement to maintain a high-quality standard [18,19]. As a result, the topic has aroused the curiosity of researchers in recent decades. Hiemenz [20] and Homann [21] were among the earliest to discuss the classic two-dimensional stagnation point problem. Since then, some researchers have undertaken numerous studies on stagnation point flow within the diverse flow and coordinate systems. Kumari and Nath [22] used the boundary layer theory to invigilate the mixed convection stagnation point flow in Maxwell fluids employing the finite difference method. Halim et al. [23] examined the nanoparticle’s active–passive parameters in the stagnation region of Maxwell fluid using a shooting method. They found that the stagnation parameter mutually improves the nanofluid’s heat transfer performance in active–passive schemes.

Nanofluids have become even more essential than they have been for the past two decades as a result of growing demands in developing effective solutions to enhance heating devices. In this modern-day landscape, heat transfer systems are found in almost every industry, including heat exchangers, computer processors, solar collectors, aerospace technology, and medical drug carriers [24,25,26,27]. Gupta et al. [24] and Chamsa-ard et al. [25] discussed the applications as well as some of the barriers and challenges related to maximising nanofluid potential. Future opportunities are also identified to accomplish the nanofluid vision. Additionally, Babu et al. [26], and Huminic and Huminic [27] reviewed a new class of modern fluid known as hybrid nanofluids. They reported that hybrid nanofluids are capable of significantly improving thermal conductivity in heat exchangers.

Nanofluids are a cleverly designed admixture of traditional fluid that use a limited amount of nanoparticles that assist in enhancing the thermal capabilities of convectional fluids. Choi and Eastman [28], who initially projected the concept of nanoparticles dispersion in a base fluid, are responsible for the practical success in this area. Later on, hybrid nanofluids were introduced to support the optimisation of the thermal system by dispersing several nanoparticles in a base fluid. Suresh et al. [29] employed varied nanocomposite powder concentrations to study the impact of Al_2_O_3_–Cu/H_2_O throughout their experimental investigation. Compared to pure water and conventional nanofluid, they discovered that the suspension of Al_2_O_3_–Cu hybrid nanoparticles improves heat transfer performance in a straight, circular tube. In another study, Takabi and Shokouhmand [30] concluded that using hybrid nanofluids enhances the heat transfer rate as compared to pure water and nanofluids, but it has a negative effect on the friction factor and appears to be significantly offset by the pressure drop loss. Nowadays, a significant number of studies on hybrid nanofluids have been published by past scholars, including Zainal et al. [31], Khashi’ie et al. [32], Waini et al. [33], and Algehyne et al. [34].

According to the studies mentioned above and a thorough analysis of the literature, no research on the boundary layer flow and heat transfer of Maxwell hybrid nanofluids in the stagnation area has been performed to date. Thus, the numerical computations for the mathematical model of boundary layer flow and heat transfer in the stagnation region employing the Maxwell hybrid nanofluids are considered in this study. The bvp4c technique in the MATLAB platform has been used to solve the specified problem in this study. Since several solutions are present, a stability analysis is conducted to demonstrate the physical interpretation of the obtained solutions.

## 2. Mathematical Model

**Figure 1:**
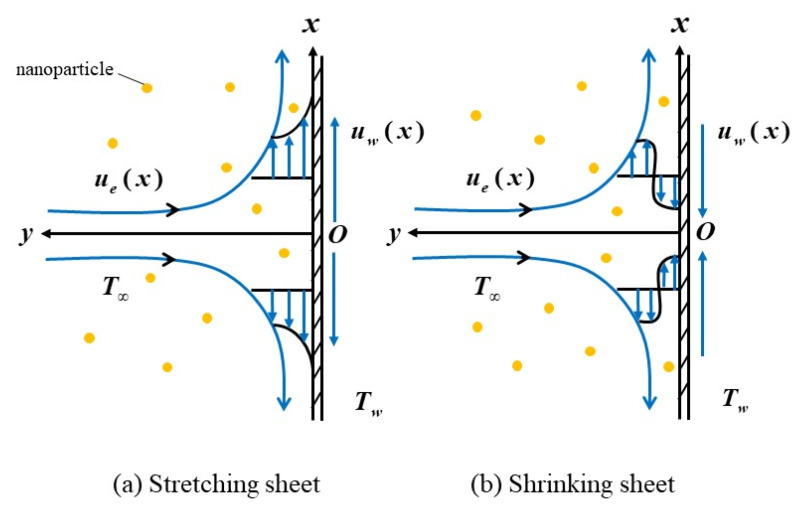
The geometrical coordinates and flow pattern for (**a**) stretching sheet and (**b**) shrinking sheet.

Figure 1 illustrates the geometrical coordinates and flow patterns of steady stagnation point flow in Maxwell hybrid nanofluids towards a stretching/shrinking surface. The stretching velocity is *u_w_*(*x*) = *cx*, in which *c* > 0 and *c* < 0 denote the stretching and shrinking constant variable, respectively, whereas the velocity of the ambient fluid is remarked as *u_e_*(*x*) = *ax*. Additionally, the sheet does not move when c=0. The temperature of the surface takes the constant value $T_{w}$, while the ambient temperature is $T_{\infty }$. Now, the respective problems can be modelled by [5,6]

(1) $$\frac{\partial u}{\partial x}+\frac{\partial v}{\partial y}=0,$$

(2) $$u\frac{\partial u}{\partial x}+v\frac{\partial u}{\partial y}=u_{e}\frac{du_{e}}{dx}+\frac{\mu_{hnf}}{\rho_{hnf}}\frac{\partial^{2}u}{\partial y^{2}}-k_{0}\left(u^{2}\frac{\partial^{2}u}{\partial x^{2}}+v^{2}\frac{\partial^{2}u}{\partial y^{2}}+2uv\frac{\partial^{2}u}{\partial x\partial y}\right),$$

(3) $$u\frac{\partial T}{\partial x}+v\frac{\partial T}{\partial y}=\frac{k_{hnf}}{{\left(\rho C_{p}\right)}_{hnf}}\frac{\partial^{2}T}{\partial y^{2}}-\frac{1}{{\left(\rho C_{p}\right)}_{hnf}}\frac{\partial q_{r}}{\partial y},$$

subject to

(4) $$\begin{array}{l} u=u_{w}\left(x\right),\;v=0,\;T=T_{w}\;\mathrm{at}\;y=0,\\ u\to u_{e}\left(x\right),\;T\to T_{\infty }\;\mathrm{as}\;y\to \infty . \end{array}$$

Note that $\mu_{hnf}$ is the dynamic viscosity of Maxwell hybrid nanofluids, T is the temperature of Maxwell hybrid nanofluids, $k_{hnf}$ is the Maxwell hybrid nanofluids heat/thermal conductivity, and $\rho_{hnf}$ and ${\left(\rho C_{p}\right)}_{hnf}$ are the Maxwell hybrid nanofluids density and the heat capacity, respectively. Table 1 showed characteristic properties [35,36] of the hybrid nanofluids used in this study, where ρ stand for density, and k and $C_{p}$ indicate thermal conductivity and heat capacity constant pressure, respectively. Next, Table 2 demonstrates the nanoparticles characteristic properties [37] symbolised by $\phi_{1}$ Cu (copper) and $\phi_{2}$ Al_2_O_3_ (alumina) together with SA (sodium alginate) pronounced as base fluid. It is worth mentioning that there are a few significant limitations to this study. First, those properties are only applicable to spherical nanoparticles and are not available to other nanoparticle forms. Second, since the hybrid Al_2_O_3_-Cu/H_2_O nanofluids is assumed stable, the influence of stabilizers is not taken into account in this study. As a result, the aggregation and sedimentation effect towards the hybrid nanofluids is omitted.

$\rho_{hnf}=\left(1-\phi_{hnf}\right)\rho_{f}+\phi_{1}\rho_{s1}+\phi_{2}\rho_{s2}$$\mu_{hnf}=\frac{\mu_{f}}{{\left(1-\phi_{hnf}\right)}^{2.5}}$${\left(\rho C_{p}\right)}_{hnf}=\left(1-\phi_{hnf}\right){\left(\rho C_{p}\right)}_{f}+\phi_{1}{\left(\rho C_{p}\right)}_{s1}+\phi_{2}{\left(\rho C_{p}\right)}_{s2}$$\frac{k_{hnf}}{k_{f}}=\left[\frac{\left(\frac{\phi_{1}k_{s1}+\phi_{2}k_{s2}}{\phi_{hnf}}\right)+2k_{f}+2\left(\phi_{1}k_{s1}+\phi_{2}k_{s2}\right)-2\phi_{hnf}k_{f}}{\left(\frac{\phi_{1}k_{s1}+\phi_{2}k_{s2}}{\phi_{hnf}}\right)+2k_{f}-\left(\phi_{1}k_{s1}+\phi_{2}k_{s2}\right)+\phi_{hnf}k_{f}}\right]$.

We now utilise the Rosseland approximation [38], hence

(5) $$q_{r}=-\frac{4\sigma^{*}}{3k^{*}}\frac{\partial T^{4}}{\partial y},$$

where $\sigma^{*}$ and $k^{*}$ are the Stefan–Boltzmann and mean absorption coefficients, respectively. Next, consider extending Taylor’s series to the temperature difference $T^{4}$ in the flow. As a result, by extending $T^{4}$ over $T_{\infty }$ while neglecting the higher-order terms, we have

(6) $$T^{4}≅4T_{\infty }{}^{3}T-3T_{\infty }{}^{4},$$

so that

(7) $$\frac{\partial q_{r}}{\partial y}=-\frac{16T_{\infty }{}^{3}\sigma^{*}}{3k^{*}}\frac{\partial^{2}T}{\partial y^{2}}.$$

Employing Equation (7) into (3), we have

(8) $$\frac{\partial T}{\partial t}+u\frac{\partial T}{\partial x}+v\frac{\partial T}{\partial y}=\frac{1}{{\left(\rho C_{p}\right)}_{hnf}}\left(k_{hnf}+\frac{16T_{\infty }{}^{3}\sigma^{*}}{3k^{*}}\right)\frac{\partial^{2}T}{\partial y^{2}}.$$

As in Ramesh et al. [5], the following similarity variable is obtained:

(9) $$\begin{array}{l} \psi =\sqrt{a\nu_{f}}xf\left(\eta \right),\;\eta =y\sqrt{a/\nu_{f}},\;\theta \left(\eta \right)=\frac{T-T_{\infty }}{T_{w}-T_{\infty }},\\ u=axf^{\prime }\left(\eta \right),\;v=-{\left(a\nu_{f}\right)}^{1/2}f\left(\eta \right). \end{array}$$

Substituting the similarity variables (9) into Equations (2) and (8), the appropriate mathematical models for the present problem are

(10) $$\frac{\mu_{hnf}/\mu_{f}}{\rho_{hnf}/\rho_{f}}f^{\prime \prime \prime }+ff^{\prime \prime }-{f^{\prime }}^{2}-\left(f^{2}f^{\prime \prime \prime }-2ff^{\prime }f^{\prime \prime }\right)K+1=0,$$

(11) $$\frac{1}{\mathrm{Pr}}\left(\frac{1}{{\left(\rho C_{p}\right)}_{hnf}/{\left(\rho C_{p}\right)}_{f}}\right)\left(\frac{k_{hnf}}{k_{f}}+\frac{4}{3}Rd\right)\theta^{\prime \prime }+f\theta^{\prime }=0,$$

(12) $$\begin{array}{c} f\left(0\right)=0,\;f^{\prime }\left(0\right)=\lambda ,\;\theta \left(0\right)=1,\\ f^{\prime }\left(\eta \right)\to 1,\;\theta \left(\eta \right)\to 0\;\mathrm{as}\;\eta \to \infty . \end{array}$$

From the equations above, $\mathrm{Pr}=\nu_{f}/\alpha_{f}$ is the Prandtl number, $Rd=4\sigma^{*}T_{\infty }{}^{3}/k_{f}k^{*}$ is the radiation parameter, $K=ak_{0}$ is the Maxwell parameter, and λ=c/a is the stretching/shrinking parameter. The physical quantities of interest in the present work are defined as

(13) $$C_{fx}=\frac{\mu_{hnf}}{\rho_{f}u_{e}{}^{2}}\left(\frac{\partial u}{\partial y}\right),\;Nu_{x}=\frac{xk_{hnf}}{k_{f}\left(T_{w}-T_{\infty }\right)}{\left(\frac{\partial T}{\partial y}\right)}_{y=0}+x{\left(q_{r}\right)}_{y=0}.$$

Note that $C_{f}$ is the skin friction coefficient and $Nu_{x}$ is the local Nusselt number. Utilising (9) and (13), we have

(14) Rex1/2Cf=μhnfμff″(0), Rex−1/2Nux=−(khnfkf+43Rd)θ′(0).

## 3. Analysis of Solution Stability

The stability analysis is used to evaluate the dual solutions in order to determine whether the supplied solutions are stable or otherwise. Due to the nonlinearity of the differential equations and the variability of geometric or fluid mechanical properties, the solutions of the similarity equations do not have to be unique for specified initial and boundary conditions. This can cause the solution to bifurcate, resulting in multiple solutions. Some of these solutions have physical relevance or are stable, while others are unstable. As a result, a stability analysis must be performed to determine which solution is stable or physically dependable [39,40]. Now, a dimensionless variable Γ is introduced in the following way:

(15) $$\begin{array}{l} u=ax\frac{\partial f}{\partial \eta }\left(\eta ,\Gamma \right),\;v=-{\left(a\nu_{f}\right)}^{1/2}f\left(\eta ,\Gamma \right),\;\eta =y\sqrt{a/\nu_{f}},\;\\ \theta \left(\eta ,\Gamma \right)=\frac{T-T_{\infty }}{T_{w}-T_{\infty }},\Gamma =at. \end{array}$$

Consider the unsteady flow for Equations (10) and (11); by utilizing Equation (15), we may now obtain

(16) $$\frac{\mu_{hnf}/\mu_{f}}{\rho_{hnf}/\rho_{f}}\frac{\partial^{3}f}{\partial \eta^{3}}+f\frac{\partial^{2}f}{\partial \eta^{2}}-{\left(\frac{\partial f}{\partial \eta }\right)}^{2}+\left(2f\frac{\partial f}{\partial \eta }\frac{\partial^{2}f}{\partial \eta^{2}}-f^{2}\frac{\partial^{3}f}{\partial \eta^{3}}\right)K-\frac{\partial^{2}f}{\partial \eta \partial \Gamma }+1=0,$$

(17) $$\frac{1}{\mathrm{Pr}}\left(\frac{1}{{\left(\rho C_{p}\right)}_{hnf}/{\left(\rho C_{p}\right)}_{f}}\right)\left(\frac{k_{hnf}}{k_{f}}+\frac{4}{3}Rd\right)\frac{\partial^{2}\theta }{\partial \eta^{2}}+f\frac{\partial \theta }{\partial \eta }-\frac{\partial \theta }{\partial \Gamma }=0,$$

(18) $$\begin{array}{l} f\left(0,\Gamma \right)=0,\;\frac{\partial f}{\partial \eta }\left(0,\Gamma \right)=\lambda ,\;\theta \left(0,\Gamma \right)=1,\\ \frac{\partial f}{\partial \eta }\left(\eta ,\Gamma \right)\to 1,\;\theta \left(\eta ,\Gamma \right)\to 0\;\mathrm{as}\;\eta \to \infty . \end{array}$$

The steady flow solutions can then be investigated, where $f\left(\eta \right)=f_{0}\left(\eta \right)\;\mathrm{and}\;\theta \left(\eta \right)=\theta_{0}\left(\eta \right)$:

(19) $$\begin{array}{c} f\left(\eta ,\Gamma \right)=f_{0}\left(\eta \right)+e^{-\gamma \Gamma }H\left(\eta \right),\\ \theta \left(\eta ,\Gamma \right)=\theta_{0}\left(\eta \right)+e^{-\gamma \Gamma }I\left(\eta \right), \end{array}$$

is introduced following the framework by Weidman et al. [40]. Next, Equation (13) is used to obtain the eigenvalue problems of Equations (16) and (17). Based on Equation (13), H(η) and I(η) are relatively small to $f_{0}\left(\eta \right)\;\mathrm{and}\;\theta_{0}\left(\eta \right)$, whereas γ signifies the eigenvalue. Substituting Equation (19) into Equations (16)–(18),

(20) $$\begin{array}{l} \frac{\mu_{hnf}/\mu_{f}}{\rho_{hnf}/\rho_{f}}\frac{\partial^{3}H}{\partial \eta^{3}}+f_{0}\frac{\partial^{2}H}{\partial \eta^{2}}+H\frac{\partial^{2}f_{0}}{\partial \eta^{2}}-2\frac{\partial f_{0}}{\partial \eta }\frac{\partial H}{\partial \eta }+\gamma \frac{\partial H}{\partial \eta }\\ \;-\left(f_{0}{}^{2}\frac{\partial^{3}H}{\partial \eta^{3}}-2f_{0}H\frac{\partial^{3}f_{0}}{\partial \eta^{3}}-2\left(f_{0}\frac{\partial f_{0}}{\partial \eta }\frac{\partial^{2}H}{\partial \eta^{2}}+f_{0}\frac{\partial H}{\partial \eta }\frac{\partial^{2}f_{0}}{\partial \eta^{2}}+H\frac{\partial f_{0}}{\partial \eta }\frac{\partial^{2}f_{0}}{\partial \eta^{2}}\right)\right)K=0, \end{array}$$

(21) $$\frac{1}{\mathrm{Pr}}\left(\frac{1}{{\left(\rho C_{p}\right)}_{hnf}/{\left(\rho C_{p}\right)}_{f}}\right)\left(\frac{k_{hnf}}{k_{f}}+\frac{4}{3}Rd\right)\frac{\partial^{2}I}{\partial \eta^{2}}+f_{0}\frac{\partial I}{\partial \eta }+H\frac{\partial \theta_{0}}{\partial \eta }+\gamma I=0,$$

(22) $$H\left(0\right)=0,\frac{\partial H}{\partial \eta }\left(0\right)=0,I\left(0\right)=0,\frac{\partial H}{\partial \eta }\left(\eta \right)\to 0,I\left(\eta \right)\to 0.$$

Following that, we define $f_{0}\left(\eta \right)\;\mathrm{and}\;\theta_{0}\left(\eta \right)$ to be the steady-state flow’s solutions, which were carried out by Γ→0. The solution to the linear eigenvalue problem is eventually discovered as

(23) $$\begin{array}{l} \left(\frac{\mu_{hnf}/\mu_{f}}{\rho_{hnf}/\rho_{f}}\_Kf_{0}{}^{2}\right)H^{\prime \prime \prime }+f_{0}H^{\prime \prime }-\left(2f_{0}{}^{\prime }-\gamma \right)H^{\prime }+f_{0}{}^{\prime \prime }H\\ \;+\left(f_{0}f_{0}{}^{\prime }H^{\prime \prime }+f_{0}f_{0}{}^{\prime }H^{\prime }-\left(f_{0}f_{0}{}^{\prime \prime \prime }-f_{0}{}^{\prime }f_{0}{}^{\prime \prime }\right)H\right)2K=0. \end{array}$$

(24) $$\frac{1}{\mathrm{Pr}}\left(\frac{1}{{\left(\rho C_{p}\right)}_{hnf}/{\left(\rho C_{p}\right)}_{f}}\right)\left(\frac{k_{hnf}}{k_{f}}+\frac{4}{3}Rd\right)I^{\prime \prime }+f_{0}I^{\prime }+\gamma I+\theta_{0}{}^{\prime }H=0,$$

(25) $$\begin{array}{l} H^{\prime }\left(0\right)=0,\;H\left(0\right)=0,\;I\left(0\right)=0,\\ H^{\prime }\left(\eta \right)\to 0,\;I\left(\eta \right)\to 0. \end{array}$$

In relation to [41], by loosening a boundary condition, the potential eigenvalues can be determined. At this point, $H^{\prime }\left(\eta \right)\to 0$ as η→∞ in Equation (25) is replaced with $H^{\prime \prime }\left(0\right)=1.$

## 4. Discussion

This section displays and discusses the findings of this study, including the skin friction coefficient, the local Nusselt number, the boundary layer velocity, and the temperature variation by plotting against the incorporated parameters. The bvp4c function (a built-in collocation method) is used to solve the coupled Equations (10) and (11) with numerical constraints (12). The accuracy of the results was verified with the previously reported data in Table 3. Since there are currently no experimental data found in the literature, previous numerical results are used to generate the findings of this study. The range of values for the Maxwell parameter, which is 0.10≤K≤0.13, is selected based on the results published by Lok et al. [7]. Meanwhile, the nanoparticle volume concentration parameter is restricted to $0.00\leq \phi_{2}\leq 0.03$ and the thermal radiation parameter is specified to 1.0≤Rd≤2.0 based on the work by Nayak et al. [11], and Hayat and Qasim [15], respectively. All of the abovementioned ranges are specified to ensure the existence of the dual solution, implying that the aim of this study is achievable.

A stability analysis is significant to this study as two possible solutions exist. The first solution is generally referred to as dependable as it fits the far-field boundary criterion. Nonetheless, by performing a solution stability analysis, we may surely prove the realistic solutions. In the stability analysis technique, the smallest eigenvalue, $\gamma_{1}$ reveals the properties of the numerical results. The flow is defined as stable when the smallest eigenvalue is positive since the solutions satisfy the stabilising requirement of allowing an initial decay. However, when the smallest eigenvalue is negative, the flow is considered unstable. Table 4 demonstrates that the first solution is stable, whereas the alternative is not.

**Figure 2:**
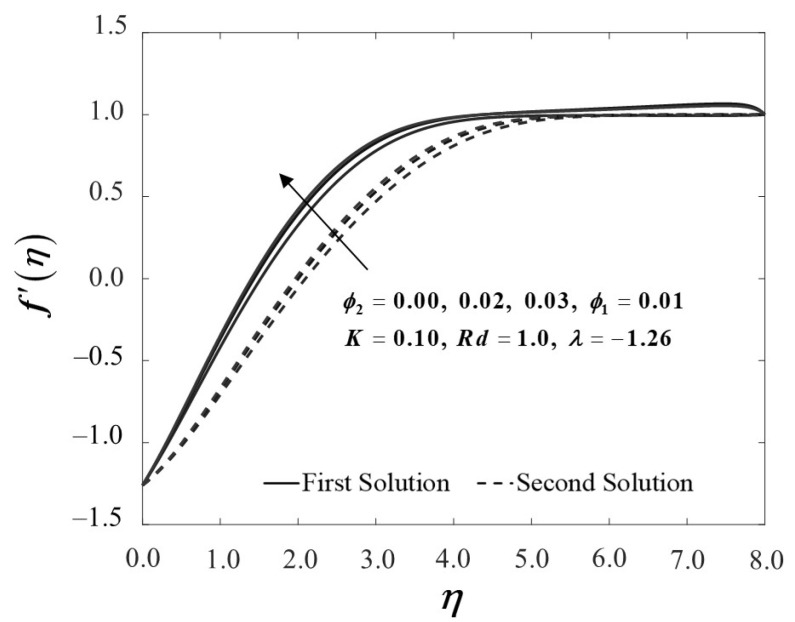
$f^{\prime }\left(\eta \right)$ towards η by assorted $\phi_{2}$.

**Figure 3:**
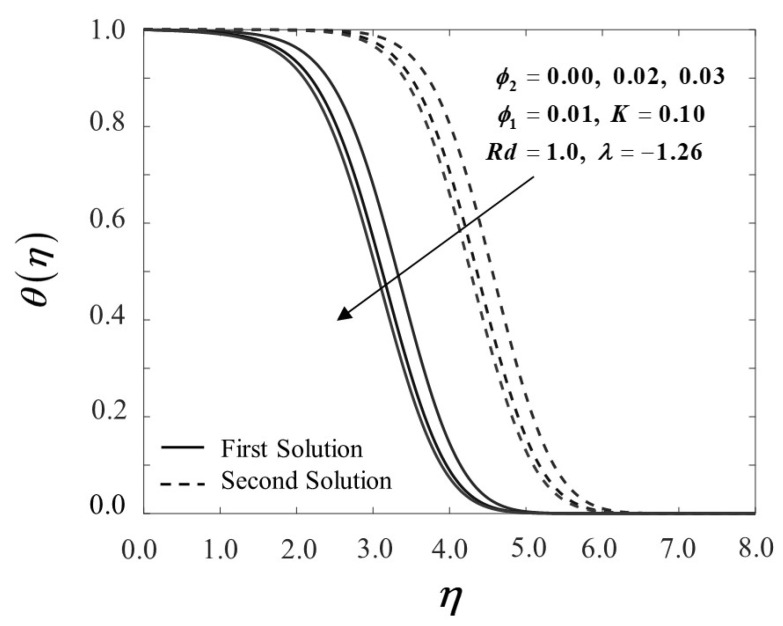
θ(η) towards η by assorted $\phi_{2}$.

Figure 2 demonstrates the influence of nanoparticle concentration on boundary layer velocity $f^{\prime }\left(\eta \right)$, whilst Figure 3 shows the temperature field profile θ(η) by adopting a dual-type of fluid, namely Al_2_O_3_/SA and Cu-Al_2_O_3_/SA. As the number of nanoparticles increases, the velocity profile inclines due to an improvement in fluid viscosity. In contrast, the presence of these nanoparticles drops the temperature profile, particularly when a dual system of nanoparticles is presented. This happens due to an increase in the mixing fluid’s thermal conductivity, hence improving the heat transfer performance. Additionally, this research is also concerned with how adding the nanoparticles into the sodium alginate, which operates as the base fluid, and affects the mechanical properties by inspecting how the skin friction coefficient $f^{\prime \prime }\left(0\right)$ and the local Nusselt number $-\theta^{\prime }\left(0\right)$ vary.

**Figure 4:**
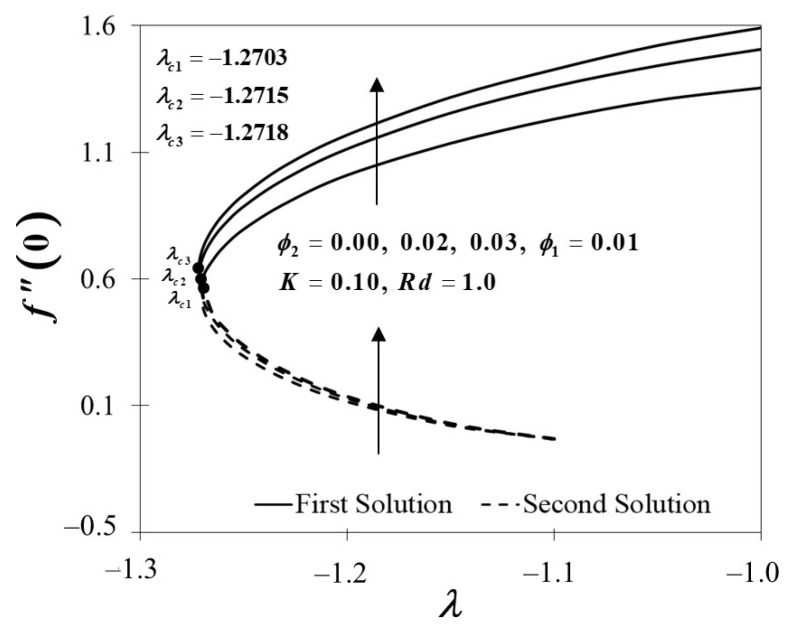
Towards λ by assorted $\phi_{2}$.

**Figure 5:**
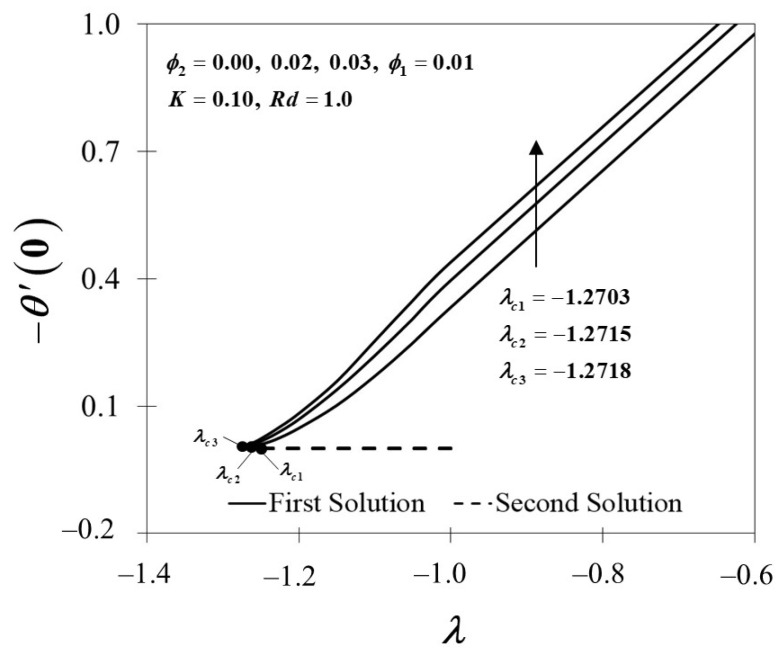
$-\theta^{\prime }\left(0\right)$ towards λ by assorted $\phi_{2}$.

Figure 4 and Figure 5 demonstrate the behaviour of $f^{\prime \prime }\left(0\right)$ and $-\theta^{\prime }\left(0\right)$ of Al_2_O_3_/SA $\left(\phi_{1}=0.01,\;\phi_{2}=0\right)$ and Cu-Al_2_O_3_/SA $\left(\phi_{1}=0.01,\;\phi_{2}=0.02,0.03\right)$. Figure 4 exhibits the improvement of $f^{\prime \prime }\left(0\right)$ as $\phi_{2}$ boosts up while the plate shrinks in both solutions. When 2% and 3% of copper volume concentration is injected, the skin friction coefficient of Cu-Al_2_O_3_/SA is higher than Al_2_O_3_/SA. From here, we note that the injection of nanoparticle concentration has raised the viscosity of Cu-Al_2_O_3_/SA. This significantly increases the fluid velocity over the surface as discussed in Figure 2. Figure 5 proves an upward trend of $-\theta^{\prime }\left(0\right)$ in the first solution, which represents the system’s cooling rate as the values of $\phi_{2}$ increase. The outcomes are consistent with the temperature profile shown in Figure 3. Consecutively, our observation reinforces the idea that a higher concentration of nanoparticles in Maxwell hybrid nanofluids improves cooling capacity over Maxwell nanofluids. Additionally, it is indeed important to note that hybrid nanoparticles have the capacity to raise heat transfer rates due to their synergistic effect. Henceforth, we may conclude that using dual types of nanoparticle concentrations in Maxwell fluid promotes effective thermal conductivity.

**Figure 6:**
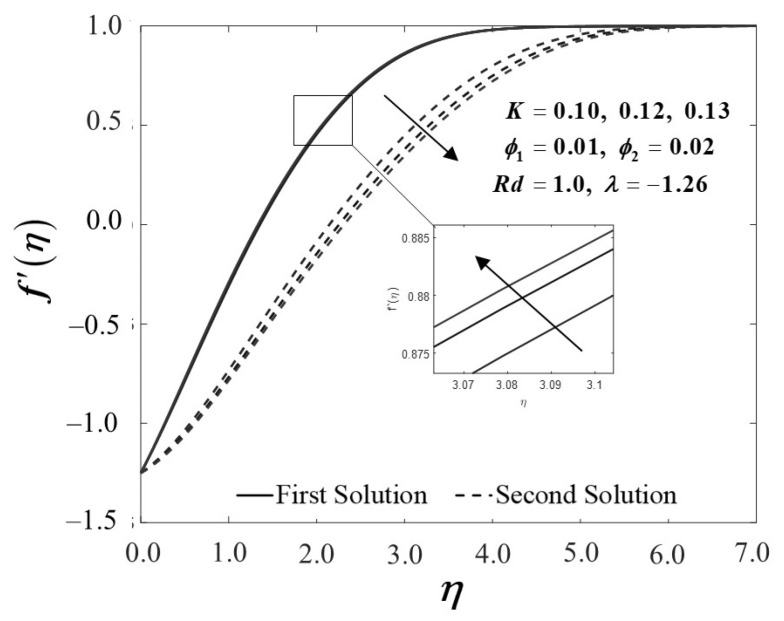
$f^{\prime }\left(\eta \right)$ towards η by assorted K.

**Figure 7:**
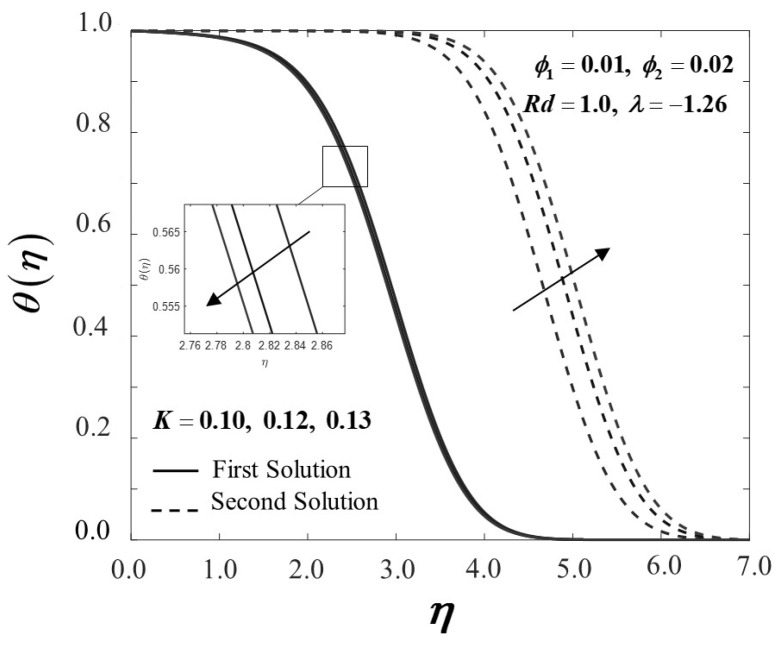
θ(η) towards η by assorted K.

The Maxwell parameter impact’s on velocity and temperature variations are shown in Figure 6 and Figure 7. As the Maxwell parameter increases, the velocity profile $f^{\prime }\left(\eta \right)$ of the first solution upsurges in Figure 6; hence, the boundary layer thickness declines. In the meantime, the velocity profile of the alternative solutions diminishes, reflecting the inclines of the boundary layer thickness. Next, as apparent in Figure 7, as the Maxwell parameter is introduced, the temperature profile θ(η) on the flat surface decreases in the first solution. Noticeably, these results are concurrent with the findings discussed by Ramesh et al. [5].

**Figure 8:**
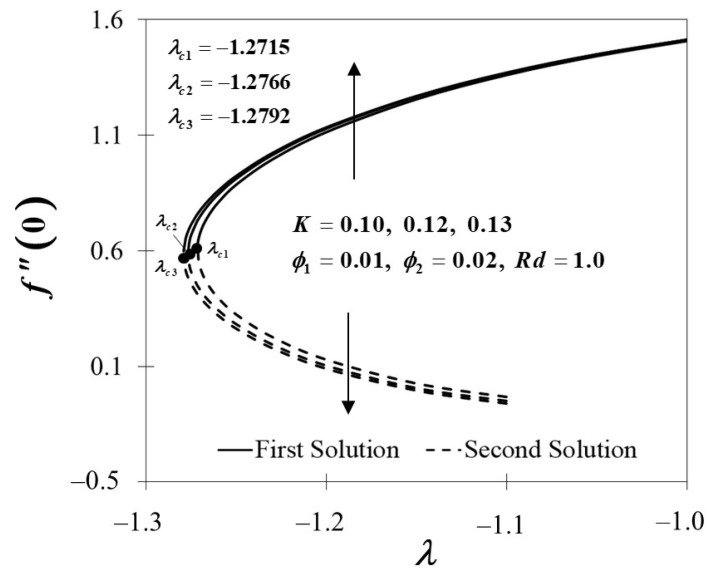
$f^{\prime \prime }\left(0\right)$ towards λ by assorted K.

**Figure 9:**
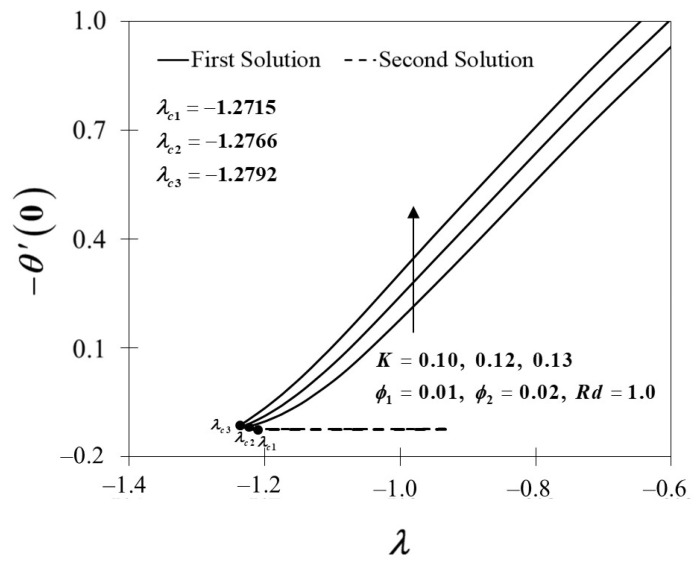
$-\theta^{\prime }\left(0\right)$ towards λ by assorted K.

The effects of the Maxwell parameter toward $f^{\prime \prime }\left(0\right)$ and $-\theta^{\prime }\left(0\right)$ is depicted in Figure 8 and Figure 9. Figure 8 shows that when the Maxwell parameter decreases, $f^{\prime \prime }\left(0\right)$ decreases, which indicates that the higher value of the Maxwell parameter leads to a higher trend of $f^{\prime \prime }\left(0\right)$. The variation of $-\theta^{\prime }\left(0\right)$ with regard to the Maxwell parameter is depicted in Figure 9. As the value of K rises, so does the value of $-\theta^{\prime }\left(0\right).$ The findings also suggest that as the value of K grows over the stagnation point flow in the Maxwell hybrid nanofluids, the thermal efficiency increases. Accordingly, increasing the Maxwell parameter values has a substantial influence on the rate of heat transfer.

**Figure 10:**
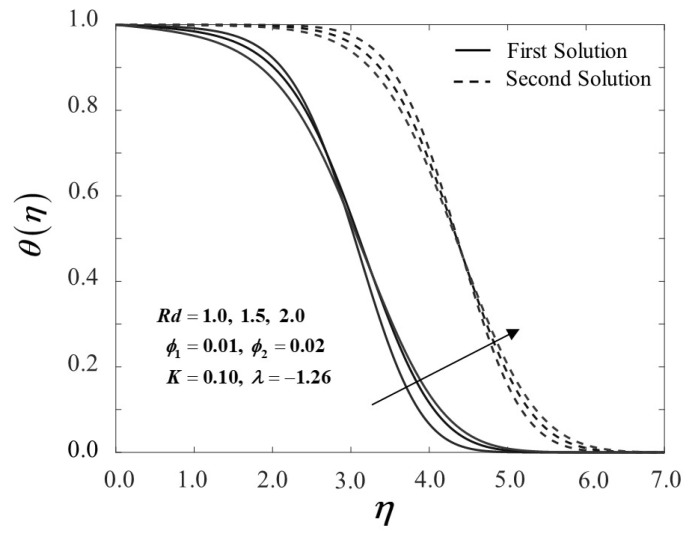
θ(η) towards η by assorted Rd.

**Figure 11:**
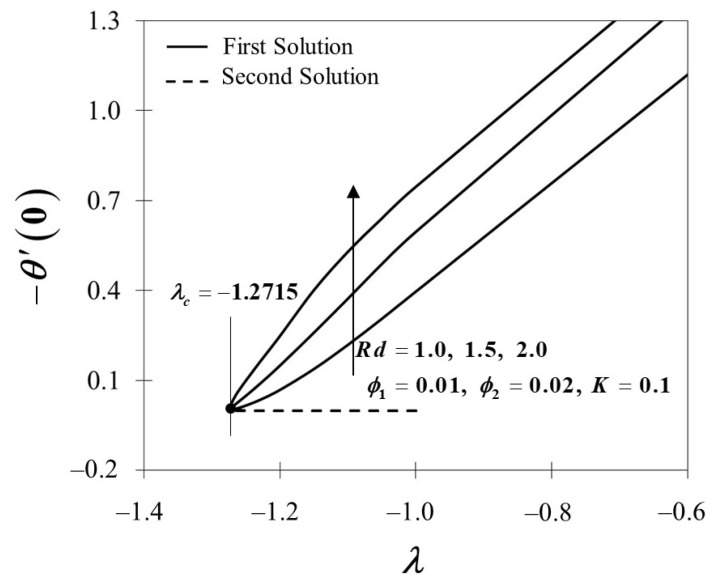
$-\theta^{\prime }\left(0\right)$ towards λ by assorted Rd.

The influence of the radiation parameter Rd on the temperature field θ(η) is illustrated in Figure 10. The temperature distribution below shows an intensification development as the radiation parameter rises. The temperature profile rises due to the growths of the conduction effects in the Cu-Al_2_O_3_/SA with the occurrence of thermal radiation. As a result, higher values of Rd imply a more heated surface. It is obvious that when the value of Rd increases, the thermal boundary layer rises. Furthermore, as shown in Figure 11, the heat transfer rate $-\theta^{\prime }\left(0\right)$ at the surface of the stagnation point flow dramatically increases as Rd grows in the current scenario. Based on these data, it is reasonable to conclude that the radiation parameter significantly impacts the thermal performance of the Maxwell hybrid nanofluids toward stagnation point flow.

## 5. Conclusions

This research presents a computational analysis of Maxwell hybrid nanofluid flows in the stagnation region with regard to heat transfer. The bvp4c procedure is used to compute the solution to the mathematical model. The significant impacts of several discussed physical parameters on non-dimensional velocity and temperature field as well as the coefficient of skin friction and local Nusselt number are depicted graphically. We draw the succeeding conclusions following a thorough examination focusing on the first solution:

- The reliability of the first solution is confirmed by the stability analysis.
- Increasing the concentration of nanoparticles in this study decreases the Maxwell hybrid nanofluids temperature and enhances the local Nusselt number, thus improving thermal conductivity.
- The velocity profile rises as the volume fraction, and the Maxwell factors improve.
- As the radiation parameter rises, the temperature profile escalates and the thickness of the momentum boundary layer tends to rise as well.
- In this particular study, the Maxwell and radiation parameters improve the local Nusselt number. Henceforward, it may be crucial to highlight that boosting these two parameters has improved the heat transfer system.

## Figures and Tables

**Table 1:** The characteristic properties [35,36].

Characteristics	Al_2_O_3_–Cu/SA
Density	$\rho_{hnf}=\left(1-\phi_{hnf}\right)\rho_{f}+\phi_{1}\rho_{s1}+\phi_{2}\rho_{s2}$
Dynamic viscosity	$\mu_{hnf}=\frac{\mu_{f}}{{\left(1-\phi_{hnf}\right)}^{2.5}}$
Thermal capacity	${\left(\rho C_{p}\right)}_{hnf}=\left(1-\phi_{hnf}\right){\left(\rho C_{p}\right)}_{f}+\phi_{1}{\left(\rho C_{p}\right)}_{s1}+\phi_{2}{\left(\rho C_{p}\right)}_{s2}$
Thermal conductivity	$\frac{k_{hnf}}{k_{f}}=\left[\frac{\left(\frac{\phi_{1}k_{s1}+\phi_{2}k_{s2}}{\phi_{hnf}}\right)+2k_{f}+2\left(\phi_{1}k_{s1}+\phi_{2}k_{s2}\right)-2\phi_{hnf}k_{f}}{\left(\frac{\phi_{1}k_{s1}+\phi_{2}k_{s2}}{\phi_{hnf}}\right)+2k_{f}-\left(\phi_{1}k_{s1}+\phi_{2}k_{s2}\right)+\phi_{hnf}k_{f}}\right]$

**Table 2:** The nanoparticles and base fluid properties [37].

Characteristics	k (W/mK)	$\tilde{\beta }\times \;10^{-5}\left(\mathrm{mK}\right)$	$\rho \;({\mathrm{kg}/m}^{3})$	$C_{p}\;\left(J/\mathrm{kgK}\right)$
Cu	400	1.67	8933	385
Al_2_O_3_	40	0.85	3970	765
SA	0.6376	99	989	4175

**Table 3:** Results of $f^{\prime \prime }\left(0\right)$ and $-\theta^{\prime }\left(0\right)$ with different λ as $\phi_{1}=\phi_{2}=K=Rd=0\;\mathrm{and}\;\mathrm{Pr}=6.2$.

λ	Lok et al. [6]	Kimiaeifar et al. [42]	Wang (2008) [43]	Present Result	
	$f^{\prime \prime }\left(0\right)$	$f^{\prime \prime }\left(0\right)$	$f^{\prime \prime }\left(0\right)$	$f^{\prime \prime }\left(0\right)$	$-\theta^{\prime }\left(0\right)$
−0.25	1.402240	1.402241	1.402240	1.402241	0.856057
−0.50	1.495670	1.495671	1.495670	1.495670	0.558412
−0.75	1.489300	1.489335	1.489300	1.489299	0.258363
−1.00	1.328820	1.328809	1.328820	1.328820	1.328820
−1.15	1.082230	-	1.082230	1.082245	0.002618
−1.20	0.932470	-	-	0.932508	0.000326
−1.2465	0.584300	-	-	0.586974	0.000000

**Table 4:** The smallest eigenvalues $\gamma_{1}$ with assorted λ.

λ	First Sol.	Second Sol.
−1.0	1.6401	−1.5264
−1.10	0.3727	−0.3856
−1.105	0.1790	−0.2238
−1.1051	0.1703	−0.2105
−1.20000	0.0869	−0.1543

## Data Availability

Not applicable.

## Nomenclature

*Roman letters*	
a,c	constant (−)
$C_{fx}$	skin friction coefficient (−)
$C_{p}$	specific heat at constant pressure $\left({J\;\mathrm{kg}}^{-1}{\;K}^{-1}\right)$
f(η)	dimensionless stream function (−)
$\left(pC_{p}\right)$	heat capacitance of the fluid $\left({J\;K}^{-1}{\;m}^{-3}\right)$
k	thermal conductivity of the fluid $\left({W\;m}^{-1}K^{-1}\right)$
$k^{*}$	mean absorption coefficient (−)
K	Maxwell parameter (−)
$Nu_{x}$	local Nusselt number (−)
Pr	Prandtl number (−)
Rd	radiati (−) on parameter
Rex	local Reynolds number (−)
t	time (s)
T	fluid temperature (K)
$T^{3},T^{4}$	difference temperature (K)
$T_{\infty }$	ambient temperature (K)
u,v	velocities component in the x− and y− directions, respectively $\left({m\;s}^{-1}\right)$
$u_{e}$	velocities of the ambient (inviscid) fluid in the x-axis $\left({m\;s}^{-1}\right)$
$u_{w}$	stretching velocity $\left({m\;s}^{-1}\right)$
x,y	Cartesian coordinates (m)
*Greek symbols*	
σ	electrical conductivity (−)
$\sigma^{*}$	Stefan-Boltzmann constant coefficient (−)
β	thermal expansion coefficient (−)
γ	eigenvalue (−)
$\gamma_{1}$	smallest eigenvalue (−)
$\phi_{1}$	nanoparticle volume fractions for Al_2_O_3_ (−)
$\phi_{2}$	nanoparticle volume fractions for Cu (−)
λ	stretching/shrinking parameter (−)
η	similarity variable (−)
θ	dimensionless temperature (−)
μ	dynamic viscosity of the fluid $\left({\mathrm{kg}\;m}^{-1}{\;s}^{-1}\right)$
ν	kinematic viscosity of the fluid $\left(m^{2}{\;s}^{-1}\right)$
ρ	density of the fluid $\left({\mathrm{kg}\;m}^{-3}\right)$
Γ	dimensionless time variable (−)
*Subscripts*	
f	base fluid (−)
nf	nanofluid (−)
hnf	hybrid nanofluid (−)
s1	solid component for Al_2_O_3_ (alumina) (−)
s2	solid component for Cu (copper) (−)
*Superscript*	
${}^{\prime }$	differentiation with respect to η (−)
*Abbreviations*	
Al_2_O_3_	alumina (−)
Cu	copper (−)
SA	sodium alginate (−)
